# Toroidal response in all-dielectric metamaterials based on water

**DOI:** 10.1038/s41598-017-07399-y

**Published:** 2017-08-25

**Authors:** Ivan V. Stenishchev, Alexey A. Basharin

**Affiliations:** 1 National University of Science and Technology (MISiS), The Laboratory of Superconducting metamaterials, 119049 Moscow, Russia; 2 National University of Science and Technology (MISiS), Department of Theoretical Physics and Quantum Technologies, 119049 Moscow, Russia

## Abstract

We experimentally demonstrate for the first time the toroidal dipolar response in metamaterials based on clusters of cylindrical dielectric particles in microwave frequency range. Instead of expensive ceramic elements we used distilled water with permittivity at room temperature is about 75, while the dielectric loss tangent is not large at frequencies up to 4 GHz. Moreover, we show all-dielectric metamaterial consisting of water box with hollow tubes which is more practical for future applications. Our findings also demonstrate that the proposed ideas can be applicable in optics with low-index dielectrics.

## Introduction

Recently the dynamic toroidal response provokes an interest due to its promising properties. In particular, the strong fields localization in subwavelength area of metamolecules, high Q- factor resonators, dynamic Aharonov-Bohm effect, the new effect of electromagnetically induced transparency in metamaterials are result of maintaining the dynamic toroidal excitation^1^. Although the toroidal dipole moment is excluded from consideration in classical electrodynamics, we need to take into account its contribution for correct description the structures with toroidal topology.

Static toroidal dipole moment was firstly predicted by Zel’dovich in 1958 for parity violation explanation in the atomic nucleus^2^, but its “second birth” was due to the dynamic toroidal response demonstration in metamaterials^3^. Its manifestation has been shown in microwave based on periodically arranged clusters of 4 SRR’s (Split Ring Resonators), in which the conductive currents were excited resembling the poloidal currents flowing along the meridians of the torus by plane electromagnetic wave^3^. This configuration provided the suppressed magnetic and electric moments, and the manifistation of the toroidal dipole in the metamolecules. It was the first demonstration of the dynamic toroidal response in metamaterials, which stimulated the progress of other metamaterials mimicking the toroidal topology^1^. We can distinguish an important disadvantage, which is complicated design of the toroidal metamolecules capable of maintaining strong toroidal excitations. Note, the development of such structures in the microwave and in the sub-terahertz is acceptable for fabrication. Although, in refs 4 and 5 the authors could fabricate a hybrid metamolecules, to our knowledge there are unique cases of the experimental toroidal response proposition in the optical range in literature. The second important limitation of conducting metamaterials, especially at the higher frequencies is the Joule losses, which can be compensated by using a superconducting inclusions or dielectrics. Low loss tangent of these materials provides advantage in all frequency ranges^6, 7^. However, high values of permittivity are achievable only at low frequencies, but for optical frequencies silicon is a promising material due to the adjusted methods of fabrication and cheapness. All-dielectric metamaterials consist of periodically arranged dielectric particles or clusters based on them^6, 7^. Mie- type resonance occurs when the wavelength inside the particle is about equal to the average between its diameter and half of its circumference. Due to the electrical permittivity of the scatterer is much higher than the permittivity of the surrounding medium, the resonance becomes very sharp and tends to coincide with the corresponding natural electromagnetic mode in the particles; this mode is associated with strong displacement currents inside the scatterer, leading to the possibility of a high electric or (and) magnetic field. In particular, optical magnetic moment, Fano-resonance in hybrid structures, negative permittivity and permeability and even toroidal response were demonstrated in all-dielectric metamaterials^6–20^. Especially we emphasize the anapole observation in a single dielectric disk in optics, which is the result of destructive interference between the toroidal and electric dipole moment^21^.

**Figure 1:**
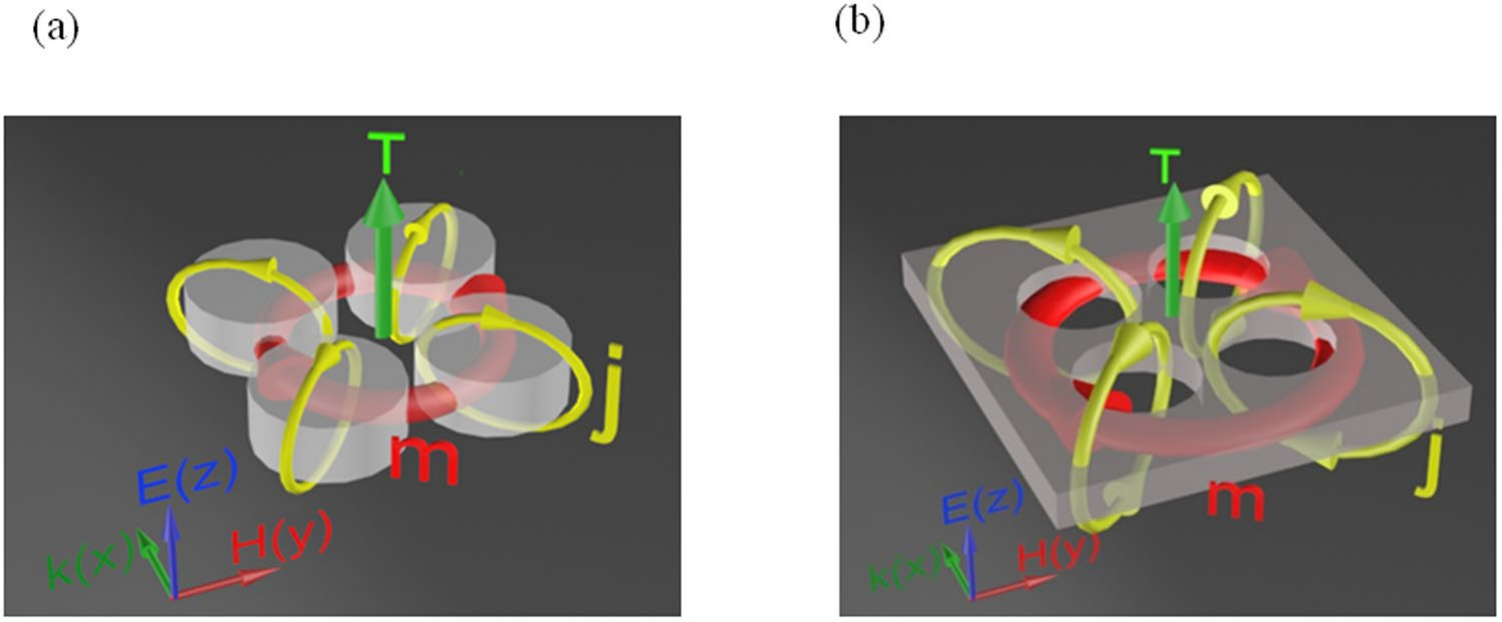
Two different types of metamolecules. (**a**) Metamolecules consist of 4 cylinders with dielectric particles. (**b**) Dielectric slab with perforated cylindrical holes. (**j** - displacement currents induced by vertically polarized plane wave, **m** - red arrows showing the magnetic field and the green arrow shows the toroidal moment **T** in metamolecules).

In this paper, we experimentally demonstrate at the first time toroidal response of all-dielectric metamaterials in microwave. Here, we propose two simplest for fabrication configurations based on all-dielectric clusters called metamolecules. The clusters of first metamaterial consist of four high-index dielectric particles (Fig. 1a). This metamaterial was investigated earlier, and theoretically demonstrated the toroidal excitation in LiTaO_3_ particle clusters in the terahertz frequency range^18^. However, for our microwave study, the water exhibits dielectric properties in microwave with low losses. At the same time, water was proved to be a candidate for dielectric metamaterials due to its convenience for metaatoms fabrication^22–24^. Clusters of the second metamaterial are the inverted version of the first one, that is the high index dielectric slab with perforated identical cylindrical holes (Fig. 1b).

## Results

### Theory

**Figure 2:**
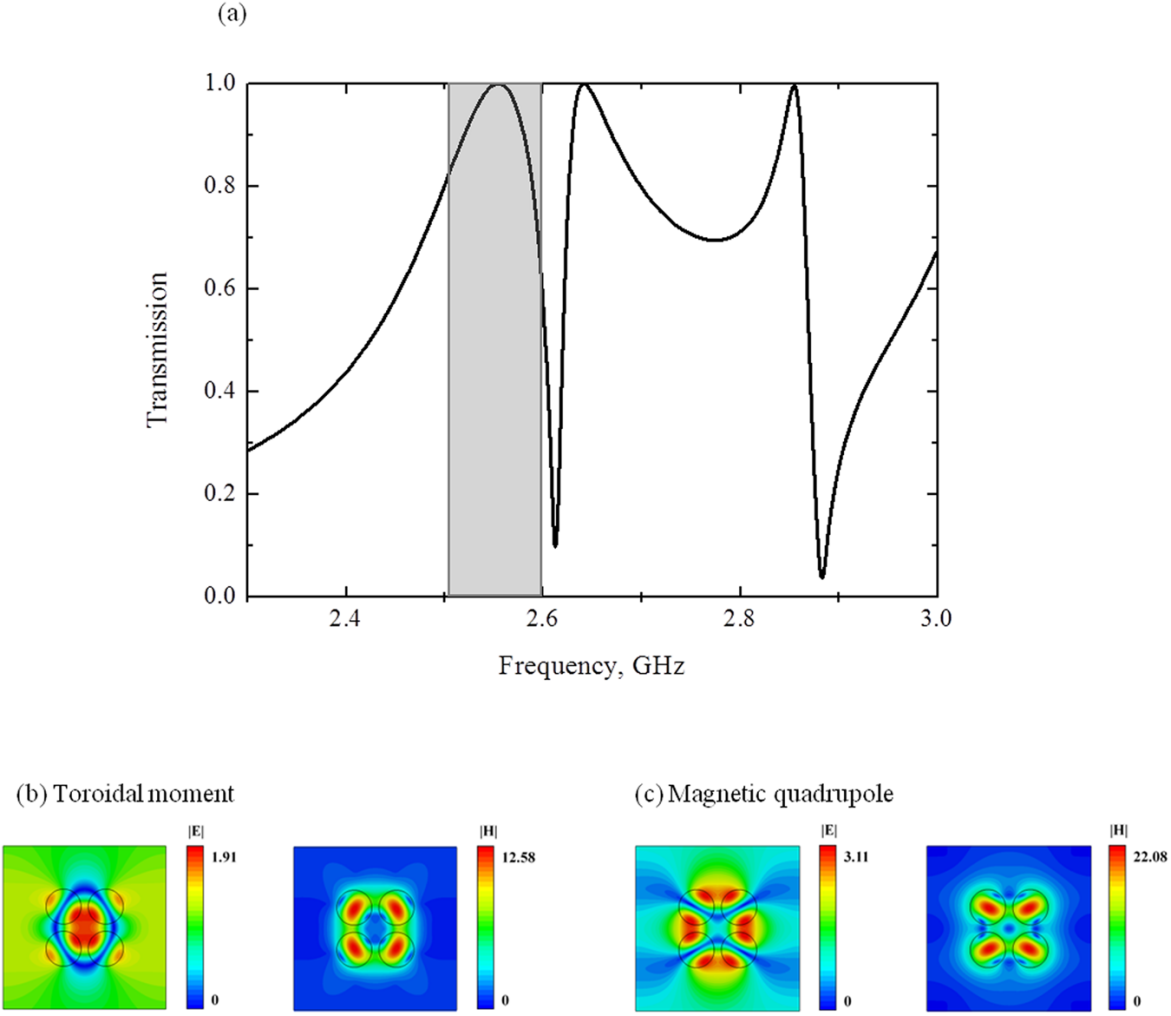
(**a**) Theoretical results of transmission calculated by HFSS for the first sample (Fig. 1a) for the lossless case, the electric and magnetic fields distribution normilized on incident wave intensity on (**b**) the toroidal frequency excitation 2.57 GHz and (**c**) the magnetic quadrupole frequency 2.67 GHz.

Let us consider the first structure of metamolecule based on four high index dielectric cylinders. We assume in our model that the cylinders are infinitely elongated, their permittivity is very close to the permittivity of water at room temperature (*ε* ~ 74) without losses. The cylinders have radius *R* = 5 mm and are arranged with the center-to-center separation *a* = 12 mm. Metamolecules are surrounded by vacuum and periodically located in ±*y* direction with step d = 45 mm and forming metamaterials slab. The infinite cylinder elongation allows to describe the electromagnetic response as a 2D model. The electromagnetic properties of metamaterial are computed by commercial Maxwell’s equation solver HFSS. The metamaterial slab is proposed by its single unit cell with applied boundary conditions. One can observe the toroidal response for such a metamaterial at around 2.5 GHz on transmission spectra (S21 parameters) (Fig. 2a). The toroidal excitation is confirmed by electric and magnetic fields distribution on this frequency (Fig. 2b). The fields clearly indicate a peculiar closed loop of magnetic vortex that penetrates all four cylinders and corresponds to the displacement poloidal currents in each cylinder. Correspondingly, the electric field exactly reproduces a hot spot in free space between cylinders as was proposed in ref. 18.

**Figure 3:**
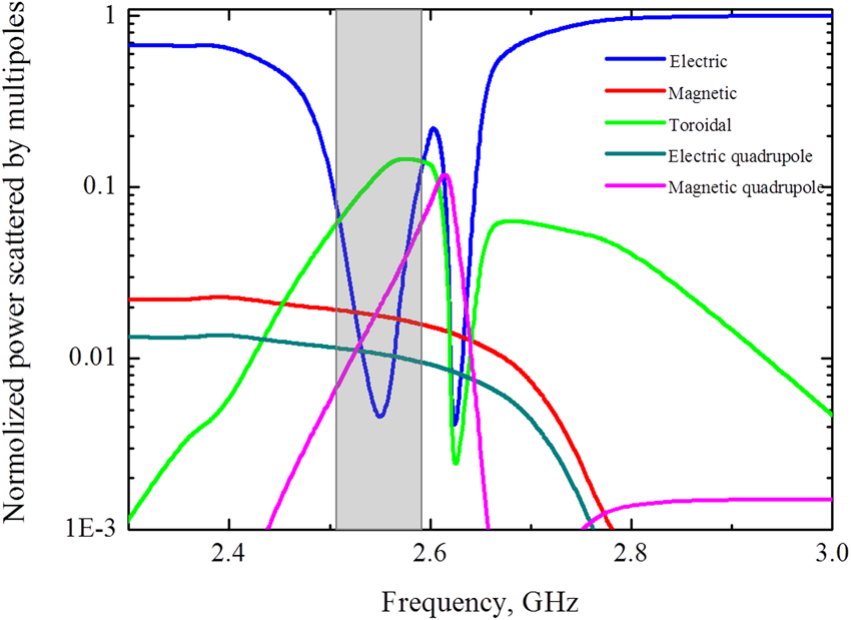
Contributions of the five strongest multipoles excited in the metamaterial array of the first sample, depicted on Fig. 1a for the lossless case.

To evaluate the role of multipoles in forming the resonant response we compare the powers scattered by 5 strongest multipoles (Fig. 3). We calculate the multipole moments induced in metamolecules based on density of displacement currents in the dielectric inclusions obtained from simulations^3^. The results of calculation are presented by electric **P**, magnetic **M**, toroidal **T** dipoles, electric **Qe** and magnetic **Qm** quadrupoles normalized to the incident power. One can observe the dominating toroidal dipole moment on the frequency 2.55 GHz around the first resonance peak and sufficiently suppressed electric dipole. While the second peak is a manifestation of magnetic quadrupole moment. We note that the dip on 2.6 GHz is strongly defined by minimum of electric and toroidal multipoles, whereas magnetic quadrupole peak on Fig. 2c is result of Fano-type resonance between magnetic quadrupole, electric dipole and close to them toroidal dipole. Electric fields for this peak at frequency 2.67 GHz is distributed between cylinders, while magnetic fields are confined inside the cylinders (Fig. 2c). Meanwhile, the fields are absent in the center of cluster.

Remarkably, such a mode maybe seen as a part of cloaked system. In particular, some particle can be placed at the center of metamolecule and, thus, hidden from distant observer on magnetic quadrupole frequency (Fig. 2c).

**Figure 4:**
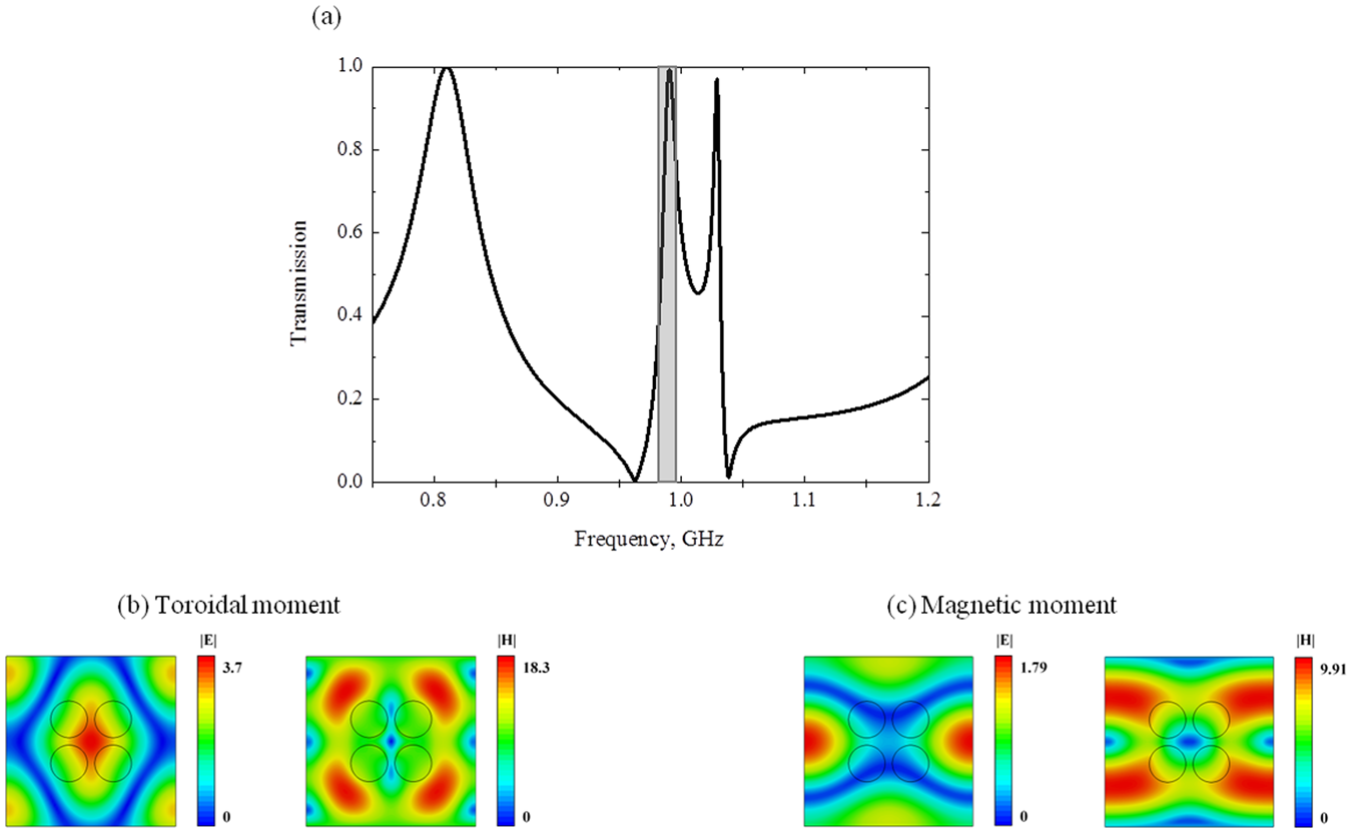
(**a**) Theoretical results of transmission calculated by HFSS for the second sample for the lossless case, the electric and magnetic fields distribution normilized on incident wave intensity on (**b**) the toroidal excitation frequency 1.09 GHz and (**c**) the magnetic excitation frequency 0.8 GHz.

Unlike the first structure, the second one represents a dielectric slab with perforated holes (Fig. 1b). The benefit of this inverted configuration is evident because of sufficient redshift of toroidal peak (Fig. 4a) more than twice due to Mie – resonance in larger parts of metamolecules. In this case the fields are concentrated around of vacuum holes with ε = 1, while their localization appears in the high-index region of metamolecules. Thus the fields are compressed in dielectric region. However, the electric field of the toroidal mode is almost concentrated in dielectric region between holes, while the magnetic field is circulating around holes (Fig. 4b).

**Figure 5:**
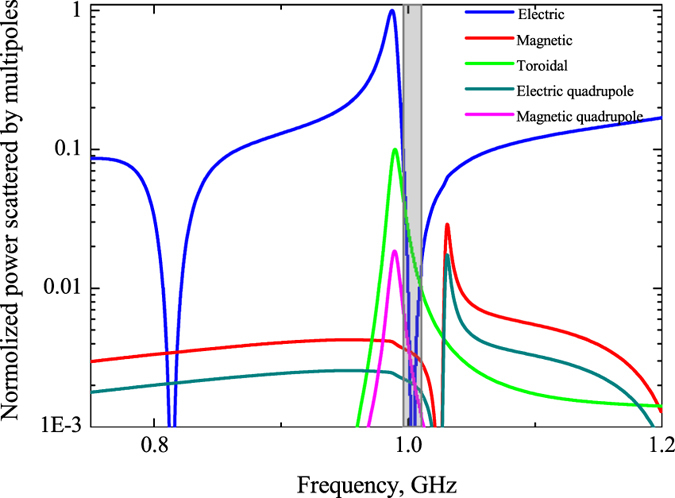
Contributions of the five strongest multipoles excited in the metamaterial array of the second sample, depicted on Fig. 1b for the lossless case.

We note that such configuration is very promising for optical and THz applications. We can expect that the *Si* dielectric slab with holes like depicted on Fig. 1b, for instance, with tunable conductivity can be operated as optical modulator pumped by extra femtosecond pulse. The result of multipole expansion resembles a toroidal mode (Fig. 5), which represents the dominating toroidal dipole moment **T**, damped electric dipole moment **P** and significantly decreased other multipoles, which are more than 10 times less than toroidal dipole moment in vicinity of 1 GHz. Interestingly, toroidal moment substantially prevails at low frequencies. However, at frequencies near 0.8 GHz magnetic moment is significant. The origin of these resonances is illustrated on Fig. 4c, where we plot the distributions of the electric and magnetic fields within the metamolecule. It is clear, that the magnetic field lines are split in two parts. While, the electric fields are concentrating mainly between clusters.

### Experiment

Water is the most nature-friendly material and can be considered as an ingredient for dielectric microwave metamaterials and prototype for future THz and optical metamaterials. The dielectric properties of water are quite promising due to high values of permittivity and relatively small loss tangent in microwave^22–24^. Distilled water is an excellent material for fundamental research of electromagnetic phenomena and tunable metamaterials, as long as the permittivity of water is a function of temperature (ε′ (T_0°C−100°C_) ≈ 90–65). Accordingly, the tunability of water metamaterials can be achieved by heating or cooling of ingredients. Furthermore, interest to water caused by the fact that we still know very little about water and its structure. In particular, it was established that water can be crystallized at high temperatures in small volumes, which is promising for water nano-particles formation^25^.

We perform an experiment in an anechoic chamber by two horn antennas methods. Two broadband antennas П6–31 (for emission and detection) were located at a distance more than 3λ from the metamaterial sample. The transmission coefficient S21 was measured by the Vector network analyzer Rohde&Schwarz ZVB20 at frequencies 0.5–3.5 GHz.

**Figure 6:**
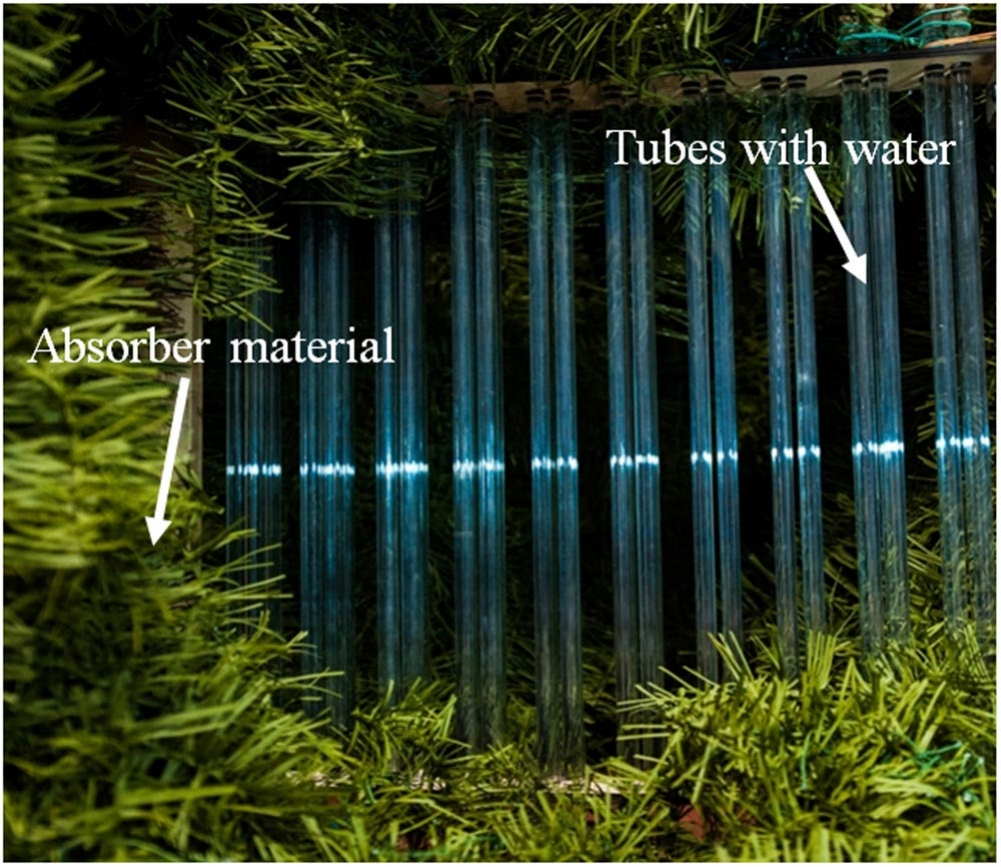
The experimental sample consists of tubes R = 5 mm radius, 1 mm thickness and 500 mm length, arranged inside the cluster with the center-to-center separation *a* = 12 mm. The period of the metamaterial is 45 mm.

To demonstrate the possibilities of the proposed metamaterial, we have fabricated the sample from the glass tubes of R = 5 mm, wall thickness = 1 mm and length = 500 mm (Fig. 6). The ends of the tubes are covered with absorber material to implement the condition of an infinite elongation metamaterial along the tubes. In the first case tubes were filled with distilled water at room temperature 20 °C, the ε^′^ = 74.5 and ε^″^ = 4.

**Figure 7:**
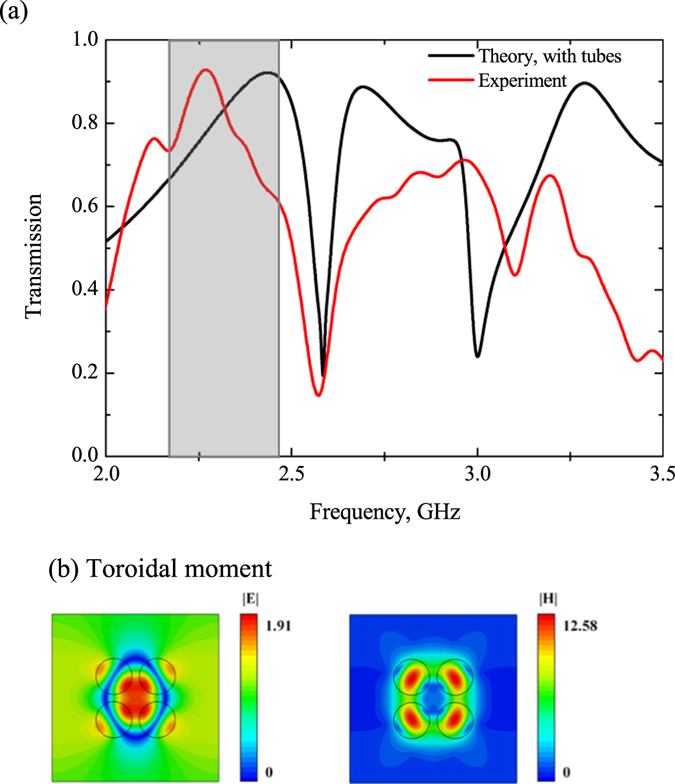
(**a**) Experimental results (Red curve) of transmission obtained in anechoic chamber for the first sample (Fig. 7a) of tubes filled up by water compared with the theoretical results (Black curve), the electric and magnetic fields distribution normilized on incident wave intensity on (**b**) the toroidal excitation frequency 2.45 GHz.

The transmission (S21) graph is depicted on Fig. 7a. We note that theoretical curve corresponds to the results of simulation considering the parameters of tubes. However, it is worth noting that the difference between theoretical and experimental data is the result of unknown permittivity of glass, but the qualitative agreement of the curves is obvious. Thus, we demonstrate here the first experimental excitation of toroidal response in proposed metamaterials. The frequency range 2–2.5 GHz corresponds to the dominating toroidal dipole moment and a dip near 2.55 GHz represents the magnetic quadrupole.

**Figure 8:**
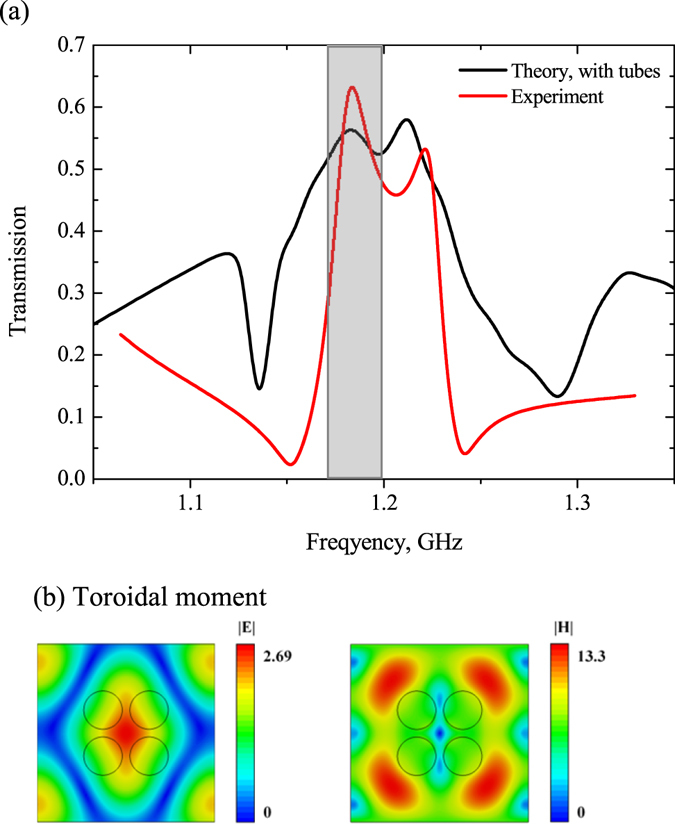
(**a**) Experimental results (Red curve) of transmission obtained in anechoic chamber for the second sample (Fig. 1b) of water box with hollow tubes compared with the theoretical results (Black curve), the electric and magnetic fields distribution normilized on incident wave intensity on (**b**) the toroidal excitation frequency 1.2 GHz.

The second sample is the plexiglass box of 440 mm × 500 mm × 44 mm filled with water, where hollow glass tubes were placed (Fig. 1b, we have not provided the picture of the sample 2, because it is impossible to perform a photo of a transparent glass tubes in the water.). In this case, we observe a significant redshift of the toroidal resonance by almost one and half times at 1 GHz compared with the response of the first structure. We stress that the unit cell is less than wavelength and constitutes ≈0.15λ. The qualitative agreement between the experimental and theoretical curves demonstrates the justification for the excitation of toroidal response in inverted metamaterial (Fig. 8a).

**Figure 9:**
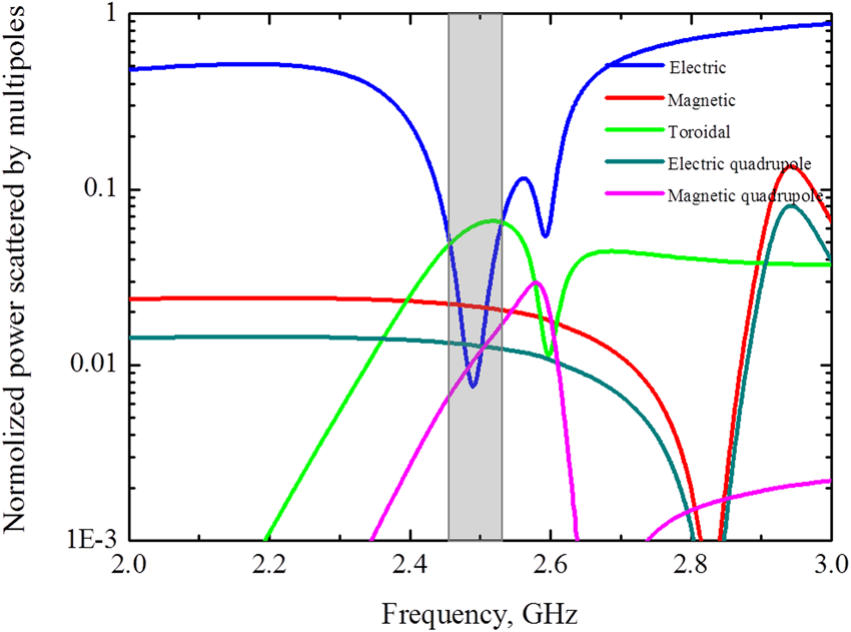
Contributions of the five strongest multipoles excited in the metamaterial array of the first sample, depicted on Fig. 1a for the loss case.

Let us estimate the difference between lossless and loss case accompanied by real dielectrics. Indeed, the toroidal transmission peak is suppressed for both structures due to the loss factor of water. It reaches 0.9 for the metamaterial based on water tubes (Fig. 7a). Nevertheless, the intensity of the toroidal moment prevails over the other multipoles in the vicinity of resonance frequency 2.45 GHz for the first structure (Fig. 9). The toroidal dipole is more than five times stronger than other multipoles. Obviously, the shape of electric field (Fig. 7b) is similar to lossless case (Fig. 2b). We observe a hot spot of electric field localized in the center of metamolecule and closed loop of magnetic vortex (Fig. 7b) with lower intensities than for lossless case. However the toroidal contribution is sufficient and experimentally confirmed (Fig. 7a).

**Figure 10:**
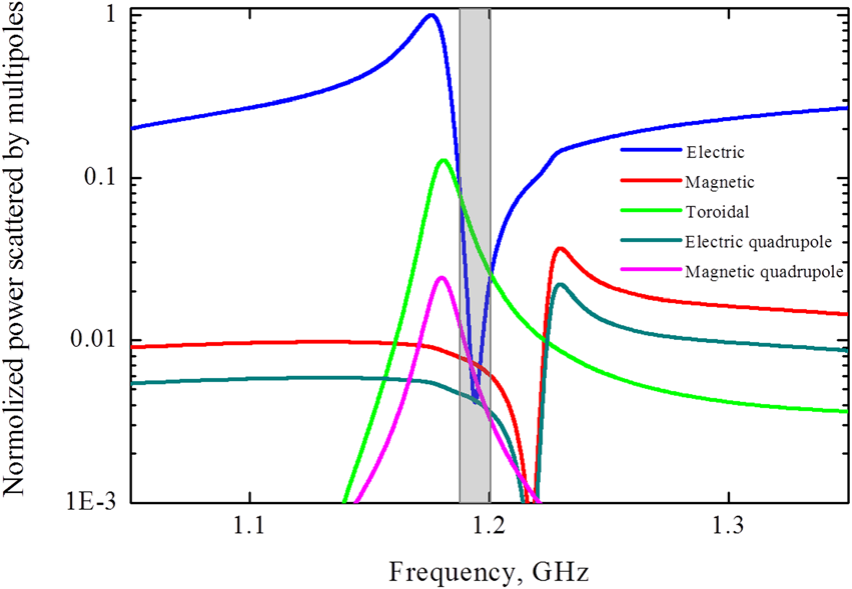
Contributions of the five strongest multipoles excited in the metamaterial slab with perforated holes depicted on Fig. 1b for the loss case.

Moreover, we consider the second structure, which has similar tendency to influence of losses as in the first structure. Indeed, the toroidal transmission peak on Fig. 8a is suppressed. Its intensity is about 0.6. In this case the electric and magnetic fields resemble the Fig. 4b differing only in amplitudes (Fig. 8b). The toroidal dipole moment exceeds other multipoles more than 5 times on the frequency 1.2 GHz (Fig. 10).

## Discussion

**Figure 11:**
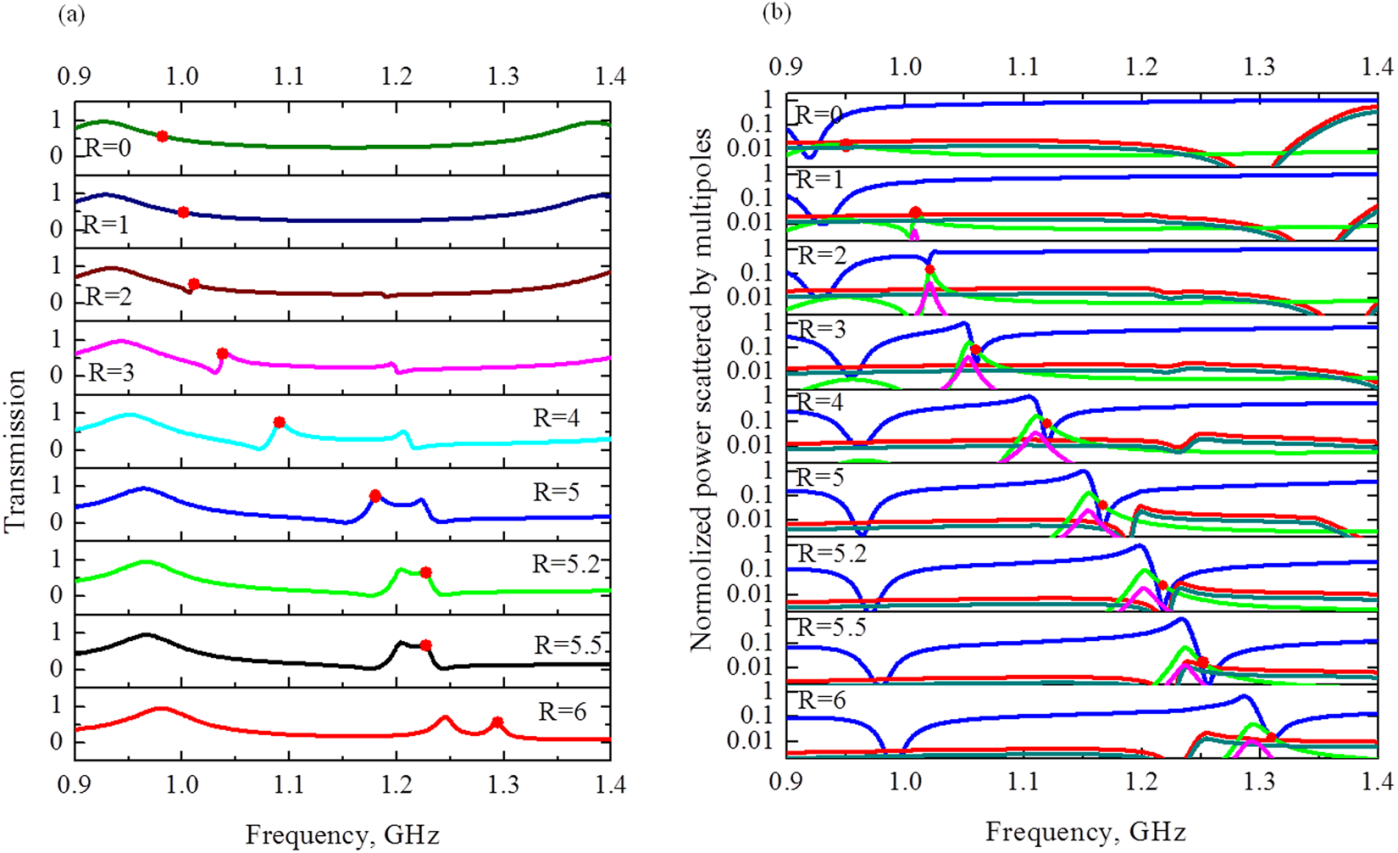
(**a**) Theoretical results of transmission for the second sample within the holes transition from R = 0 mm to R = 6 mm; (**b**) Contributions of the five strongest multipoles excited in the metamaterial based on dielectric slab with perforated holes in loss case.

Let us discuss the origin of toroidal response in the second structure. It is determined by the coupling between the Mie- magnetic modes arising in the cylinders (holes) placed in the dielectric host. One can reduce this problem to the classical Mie scattering of EM waves by the dielectric cylinders with ε_1_, embedded in medium with ε_2_, where ε_1_ < ε_2_^26^. In our case we characterize ε_1_ = 1 and ε_2_ by water permittivity (ε′ = 74.5 and ε″ = 4) at room temperature^1^. Thus, the toroidal response is determined by the hole size and dielectric permittivity of water. One can observe the resonance behavior dependence on hole radius R (Fig. 11a). Although the toroidal dipole moment is available at the all values of R, its strong contribution appears at R = 3 mm (Fig. 11b). We estimate that the resonance is blueshifted with R increasing due to transition from the classical Fabry – Perrot resonance in dielectric slab (R = 0 mm) to the evident toroidal peak (R > 3 mm). We note that Fabry –Perrot resonance is mainly characterized by the electric dipole instead of toroidal dipole moment (Fig. 11b). However, within the transition from R = 0 mm to R = 6 mm the contribution of toroidal moment is become strongest, as depicted on Fig. 11b, which is accompanied by growing of the resonant peak marked by a red spot on Fig. 11a. At the same time, the electric dipole intensity is suppressed with R increasing. Thus, the resonance is pronounced on the frequency 1.2 GHz and R = 5 mm, which is manifested by toroidal dipole moment.

The performed experiments confirmed the earlier proposed idea about the toroidal dipole excitation in all-dielectric metamaterials. However, we need to give some clarification. Although, water is the cheapest ingredient, it should be noted that it is useful only for laboratory modeling of all-dielectric metamaterials and photonic crystals^22^. Nevertheless, we would like to discuss the differences between the first and the second samples of our metamaterials. Since the fabrication of dielectric cylinders, discs or spheres^6, 7^ and even tubes^27^ for microwave and possibly even for THz range is simple, while this problem is quite complicated in the optical range. One can see that the particles should be identically produced and positioned within metamolecule in micro- and nano-scale^6, 7^.

We note that the toroidal response can be applicable to other all-dielectric metamaterials. For example, the structure based on micro-tubes of LiTaO_3_^27^ is similar to the clusters of 4 all-dielectric particles^18^ that will allow observing the toroidal response in the THz range, provided that holes are successfully drilled in cylindrical particles. In addition, the toroidal response can be observed in all-dielectric structures based on many cylinders or ellipsoids^19^.

At the same time, the second structure (Fig. 1b) is more promising due to simpler procedure of sample preparation. We just have to perforate the holes in dielectric slab. It can be possible in THz and even in optics by the FIB (Focused ion beam) method up to nm holes preparation in silicon slab for instance. Accordingly, the fabrication of micro- and nano- holes is, in our opinion, the most elegant solution for future all-dielectric optical metamaterials.

**Figure 12:**
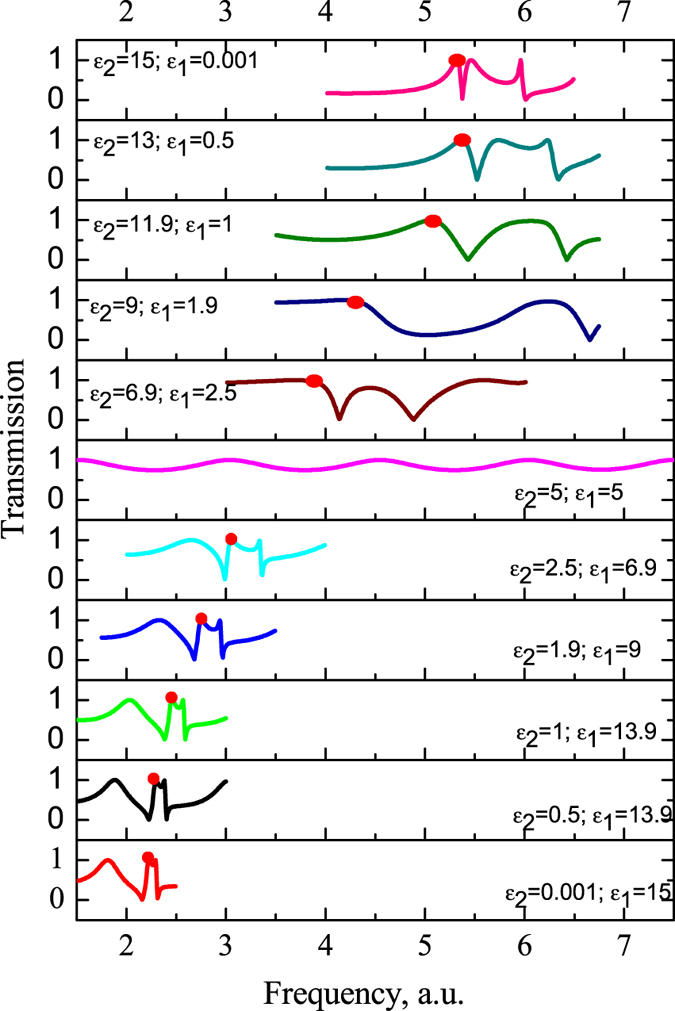
Theoretical results of transmission represents the dependence of the toroidal response of the structure’s permittivity.

Let us compare the case one (Fig. 1a) and case two (Fig. 1b) in order to demonstrate how we can use our idea in optics for low index dielectrics like silicon slab of *ε*_*1*_ permittivity with perforated holes with embedded cylinders of *ε*_*2*_. We plot a family of graphs to trace the evolution of transmission behavior depending on relation between *ε*_*1*_ and *ε*_*2*_. We pointed the toroidal response peak by red point, Fig. 12. Interestingly, the toroidal mode is characterized by the first peak for the first structure, i.e. the high index dielectric cylinders in low index matrix (*ε*_*1*_ > *ε*_*2*_), while for the second case (*ε*_*2*_ > *ε*_*1*_) the peaks have changed: the toroidal peak has become the second. Moreover we observe sufficient redshift of toroidal peak more than two times from the first graph up to last one.

Next, we discuss possible applications of proposed metamaterials. The main distinction between the first (Fig. 1a) and second metamaterials type (Fig. 1b) is the area of fields localization. In the first case the sample allows to maintain the hot spot of electric field between cylinders, then it is interesting for nonlinearities excitation and sensing of the particles placed between cylinders. At the same time the second case is promising because of hot spot localized inside the dielectric slab, one can exploit this effect for high index nonlinear phenomena without external embedded particles.

In conclusion, we proposed and experimentally studied at the first time a novel class of all-dielectric metamaterials that exhibit a resonant toroidal response in the GHz part of the spectrum. Our metamolecule is based on subwavelength clusters of two types. First sample consist of high-index dielectric cylinders arranged as four particle clusters. The second type is dielectric slab with perforated four holes clusters. We show experimentally the transmission spectra of metamaterials based on water that exhibit strong toroidal mode. Our findings can be useful for future optical design of optical all-dielectric toroidal metamaterials which can be organized and simply fabricated from the low-index dielectrics as silicon.

## Methods

### Simulations

The electromagnetic properties of the all-dielectric metamaterials are computed with the aid of a commercial Maxwell’s equation solver HFSS using the standard transient modeling approach. The simulations also provide the data of the displacement current densities in dielectrics, which is used to calculate powers radiated by conventional multipoles with toroidal dipole taken into account^3^.

### Samples fabrication

The all-dielectric samples were fabricated from glass tubes embedded in Plexiglas box.

### Microwave measurements

Transmission spectra (S21-parameter) was measured in anechoic chamber using Vector Network Analyzer Rohde&Schwarz ZVB20 connected with horn wideband antennas П6-31.
